# Discrepancies in perceptions of PTSD symptoms among veteran couples: Links to poorer relationship and individual functioning

**DOI:** 10.1111/famp.13041

**Published:** 2024-10-01

**Authors:** Kathleen M. Grubbs, Kayla C. Knopp, Chandra E. Khalifian, Elizabeth R. Wrape, Margaret‐Anne Mackintosh, Min Ji Sohn, Alexandra Macdonald, Leslie A. Morland

**Affiliations:** ^1^ Department of Veterans Affairs VA San Diego Healthcare System San Diego California USA; ^2^ Department of Psychiatry University of California San Diego California USA; ^3^ Center for Mental Health Outcomes Research Central Arkansas VA Healthcare System Little Rock Arkansas USA; ^4^ National Center for PTSD Dissemination and Training Division, VA Palo Alto Healthcare System Palo Alto California USA; ^5^ The Citadel, Military College of South Carolina Charleston South Carolina USA; ^6^ National Center for PTSD – Women's Health Science Division, VA Boston Healthcare System San Diego California USA

**Keywords:** couples, intimate relationships, post‐traumatic stress disorder, PTSD, relationship functioning, relationship satisfaction

## Abstract

Veteran and intimate partner perceptions of posttraumatic stress disorder (PTSD) may differ, and little is known about how agreement or disagreement on symptom severity is related to relationship satisfaction. Veterans and their partners (*N* = 199 couples) completed a baseline assessment for a clinical trial evaluating two couple‐based PTSD interventions. Veterans completed the PTSD Checklist for DSM‐5 (PCL‐5). Partners completed the collateral PCL‐5 (PCL‐5‐C), which asked them to rate the severity of the veteran's PTSD symptoms. Both partner and veteran completed the Couples Satisfaction Index (CSI‐32). Intraclass correlations (ICC) assessed agreement between PCL‐5 and PCL‐5‐C total and subscale scores, which was low for total PCL and for all subscales (ICC = 0.15–0.46). Actor‐Partner Interdependence Models (APIMs; actor‐only pattern) tested associations between relationship satisfaction and PTSD symptom severity (total PCL and subscales), and the magnitude and direction of difference between PCL‐5 and PCL‐5‐C (total and subscales). For veterans, more severe total PTSD and negative cognition/mood scores were associated with lower relationship satisfaction, and the direction of discrepancy for negative cognition/mood (i.e., higher veteran‐rated PTSD symptoms relative to partner's collateral report) was also associated with lower satisfaction. For partners, more severe collateral‐reported symptoms for total PTSD and all four subscales were associated with lower relationship satisfaction; further, a larger discrepancy between veterans' and partners' reports of total PTSD, negative cognition/mood, and hyperarousal were associated with lower satisfaction. These results suggest that partners may have different perceptions of PTSD symptoms, and support the potential of fostering a shared understanding of PTSD symptom severity in couples.

Extant literature supports a reciprocal negative association between posttraumatic stress disorder (PTSD) severity and relationship quality (Allen et al., 2018; Taft et al., 2011). PTSD is associated with depression (Seal et al., 2008), substance use (Burnett‐Zeigler et al., 2011; Eisen et al., 2012; McDevitt‐Murphy et al., 2010), anger (Novaco & Chemtob, 2002; Rosen et al., 2013), insomnia (Lambert et al., 2012), and sexual dysfunction (Breyer et al., 2016; Cosgrove et al., 2002; Letourneau et al., 1997), all of which can also negatively impact relationships over time. Impaired emotional intimacy and higher rates of verbal and physical aggression toward significant others and children are also common sequelae of PTSD that can further exacerbate difficulties (Monson et al., 2010; Taft et al., 2011). The association between PTSD symptoms and relationship quality is bidirectional, such that higher relationship quality and higher perceived social support can buffer against PTSD severity (Wagner et al., 2016). Higher relationship quality is associated with better treatment engagement (McGinn et al., 2017; Meis et al., 2019; Price et al., 2018) and greater symptom improvement during evidence‐based psychotherapies for PTSD (Price et al., 2013; Sayer et al., 2009; Spoont et al., 2014).

The cognitive‐behavioral interpersonal model of PTSD within a couples' context illustrates PTSD's bidirectional influence on the relationship between two members of the couple and between the dyad and the relationship milieu (Monson et al., 2010). In this model, veterans and intimate partners experience cognitions, behaviors, and emotions connected to their respective experiences of PTSD. Partner A (veteran) experiences PTSD as a set of internal cognitive symptoms (threat appraisal, difficulties with trust), behavioral symptoms (poor communication, aggression), and emotional symptoms (guilt, anger, and numbing). Partner B (significant other) simultaneously experiences their own set of cognitions (control and symptom attributions), behaviors (accommodation and distancing), and emotions (anger and sadness) in response to their partner's PTSD symptoms. Over time, this dynamic interaction between relationship partners impacts and is impacted by the larger context of the relationship including intimacy, satisfaction, consensus, and cohesion. Expanding our understanding of some key aspects of this model could help to deepen our knowledge of PTSD in a relational context (Monson et al., 2012).

One key factor at the core of the cognitive‐behavioral interpersonal model is the couple's shared understanding of the disorder itself (e.g., “Why do I/does my significant other always feel a need to monitor our surroundings?”), as well as the ways in which symptoms may affect the individual and their interpersonal dynamics, such as communication (e.g., “Why do I/does my significant other avoid sharing feelings?”). A study evaluating relationship maintenance factors (i.e., patterns within the relationship that strengthen relationship quality) found that one perception of the other partner's maintenance behaviors was important to their judgment of the overall relationship quality (Ogolsky & Bowers, 2013). A similar pattern exists for PTSD. For example, in a study of spouses of Vietnam veterans, spouses' higher ratings of PTSD severity were associated with higher levels of general distress and lower evaluations of relationship quality (Renshaw et al., 2010). Partner perceptions of withdrawal and numbing symptoms were most closely linked to relationship dissatisfaction and overall distress (Renshaw & Caska, 2012), both cross‐sectionally and within couples over time (Allen et al., 2018). Renshaw and Caska (2012) use attribution theory to explain the link, finding that partners report more distress when they interpret symptoms as threatening to the relationship (e.g., social isolation and numbing) or as related to effort, intentions, or character of their partner. Conversely, significant others report less distress when they attribute symptoms to PTSD rather than to a character trait.

Couples often differ in their perceptions of shared experiences, including their perceptions of clinical symptoms. Whereas both partners' experiences may be impacted by the effects of PTSD symptoms (e.g., communication difficulties and anger experiences; Renshaw & Caska, 2012), individuals with PTSD directly experience the internal symptoms of PTSD (e.g., intrusive memories and hyperarousal) while significant others only have external behaviors to observe, leaving significant room for interpretation. As a result, PTSD patients and their intimate partners often do not experience the presence or severity of symptoms in the same way (Calhoun et al., 2002; Gallagher et al., 1998). However, previous literature has not established whether relationship partner alignment on symptom interpretation is critical to functioning or protective against the relational impact of PTSD symptoms. Differences in partners' ratings on general relationship constructs (e.g., sociability or flexibility) have been shown to be negatively related to relationship satisfaction (Busby et al., 2001), suggesting that there may be value in promoting a shared understanding of the meaning and impact of PTSD symptoms for each member of the couple. Attribution of symptoms to PTSD on the part of intimate partners is associated with their own greater well‐being and relationship functioning (Renshaw & Caska, 2012). Thus, increasing shared significant other and veteran perceptions of PTSD symptoms could be one way to mitigate the impact of PTSD on relationships.

In sum, although the importance of significant others' perceptions of veterans' PTSD and the relevance of both partners' perspectives is well‐established, an important gap in the research exists regarding how the convergence or divergence of partners' perceptions and the magnitude of those differences are associated with relationship functioning. The goal of the current study was to investigate how veterans and their significant others view symptoms of PTSD among a sample of veteran couples seeking a couple‐based treatment for PTSD. Using guidance from Kenny et al. (2006), we first used a nomothetic approach to determine to what degree relationship partners agree or disagree on the severity of PTSD symptoms overall. Second, we used an idiographic approach to examine whether different patterns of agreement (i.e., magnitude and direction of the discrepancies between the two partners' reports) across different couples were associated with each partner's relationship satisfaction. We hypothesized that veterans and their significant others would demonstrate significant disagreement across symptoms of PTSD and that greater disagreement in symptom ratings would be associated with lower relationship satisfaction for both partners.

## METHOD

### Participants

This study included 199 dyads (*N* = 398) comprised of a veteran with PTSD or suspected PTSD based on clinician diagnosis and referral, along with their identified romantic partner. Dual‐veteran couples were eligible as long as only one partner met diagnostic criteria for PTSD. For simplicity, regardless of veteran status, the partner without a PTSD diagnosis is referred to as the “significant other,” while the PTSD‐diagnosed identified patient is referred to as the “veteran.” Eighty‐three percent of veterans in the sample were male. The mean age was 42.9 years old (*SD* = 13.9) for veterans and 41.8 (*SD* = 13.9) for significant others. As shown in Table 1, the sample of veterans was racially and ethnically diverse and representative of the VA San Diego Healthcare System. Relationship length ranged from 0.25 to 51 years (*M* = 12.9; *SD* = 12.4), with 75% of couples describing themselves as married. Couples reported having between 0 and 7 children, with an average of 1.85 (*SD* = 1.46) for significant others and 2.12 (*SD* = 1.70) for veterans.

**TABLE 1:** Sample characteristics.

Characteristics	Veteran (*n* = 199)		Significant others (*n* = 199)	
	M/n	SD/%	M/n	SD/%
Age	42.93	13.94	41.84	13.93
Gender (% male)	163	82.7	32	16.2
Race				
African American	38	19.1	30	15.1
American Indian/Alaska Native	4	2.0	3	1.5
Asian	8	4.0	12	6.0
Caucasian	118	59.3	96	48.2
Middle Eastern	2	1.0	1	0.5
Native Hawaiian/Pacific Islander	6	3.0	5	2.5
Other	13	6.5	28	14.1
Did not respond	10	5.0	24	12.1
Education				
High school graduate	139	76.8	132	73.3
College graduate	50	27.6	60	33.3
Professional/graduate school	19	10.5	24	13.3

### Procedure

Data examined for this study were cross‐sectional baseline assessment data preceding enrollment a randomized clinical trial (RCT) of brief Cognitive Behavioral Conjoint Therapy (bCBCT) for PTSD. For further study details, please refer to (Morland et al., 2022). Candidate couples were referred to a study comparing home‐based telehealth and in‐person bCBCT to in‐person PFE by the veteran's mental health provider. All dyads who met preliminary eligibility and provided informed consent completed a 3‐ to 4‐hour baseline assessment prior to being enrolled in the RCT. All veterans who completed the baseline assessment were included in the current study regardless of eventual inclusion in the RCT. The VA San Diego Healthcare System Institutional Review Board approved all methods.

### Measures

#### PTSD symptom severity

Veterans completed the PTSD Checklist (PCL‐5; Weathers et al., 2013), a 20‐item self‐report instrument with items anchored to an index trauma. Items are rated on a Likert scale from 0 to 4 based on how much the symptom has bothered the respondent in the previous month with higher scores corresponding to greater distress. Total scores are calculated by summing item values and range from 0 to 80. Items are organized into four clusters of PTSD symptoms, including intrusions, effortful avoidance, negative alterations in cognitions and mood, and alterations in reactivity symptoms, consistent with the Diagnostic and Statistical Manual of Mental Disorders—Fifth Edition criteria for PTSD (American Psychiatric Association, 2013). For veterans, the PCL‐5 has strong psychometric properties, including good internal consistency (*α* = 0.96) and test–retest reliability (*r* = 0.84; Bovin et al., 2016). In the current sample, the scale demonstrated good internal consistency for the total score (*α* = 0.911) and for subscales (*α* = 0.754–0.837).

Significant others completed the PTSD Checklist‐5‐Collateral (collateral PCL‐5 or PCL‐5‐C; Fredman et al., 2020), a 20‐item measure that focuses on the intimate partner's perception of the veteran's PTSD symptoms. The PCL‐5‐C mirrors the PCL‐5; however, the prompts are changed to, “How much has your partner been bothered by [symptom]?”. Scoring rules are identical to the PCL‐5. In this sample, the PCL‐5‐C had good internal consistency for the total score (*α* = 0.949) and for subscales (*α* = 0.797–0.905).

To operationalize the degree of agreement between PCL‐5 and PCL‐5‐C, we computed two additional variables for the total scale and for each symptom cluster subscale. First, we calculated the magnitude of the discrepancy as the absolute value of the difference between the two scores, which was then log‐transformed for normality. Because our analysis of agreement focused on only one variable (PCL) rather than a set of variables, a simple discrepancy score was the most sensible choice (Kenny et al., 2006). Second, we coded the direction of the discrepancy as 1 if the veteran's PCL‐5 score was higher than their significant other's PCL‐5‐C score ratings and 0 if the significant other's PCL‐5‐C score was higher.

#### Relationship satisfaction

Both partners completed the Couples Satisfaction Index (CSI‐32; Funk & Rogge, 2007), a self‐report measure of relationship satisfaction. Items have varied response scales and formats with each item summed to calculate a total score. The total scores ranged from 0 to 161, with higher scores indicating greater satisfaction. The CSI‐32 has demonstrated good reliability across studies, including the current sample of veterans (*α* = 0.987) and significant others (*α* = 0.976). It demonstrates convergent validity with other measures of relationship functioning (see Graham et al., 2011 for a review).

### Data analysis

#### Aim 1: Evaluating agreement between veterans and significant others on PTSD symptom severity

To investigate the agreement between the veterans' (PCL‐5) and the significant others' (PCL‐5‐C) PTSD symptom severity ratings, we calculated intraclass correlation coefficients (ICCs) and their 95% confidence intervals based on an absolute agreement two‐way mixed‐effects model. This analysis included 170 couples with a complete set of PCL‐5 and PCL‐5‐C measures. There were no significant differences between the included and excluded samples on any demographic or clinical variables of interest (e.g., PTSD severity).

#### Aim 2: Evaluating associations between PTSD Total scores and subscale scores, symptom agreement, and relationship satisfaction

To investigate whether the degree of agreement between the veteran and significant other was associated with relationship satisfaction, we ran a series of Actor‐Partner Interdependence Models (APIMs; Cook & Kenny, 2005). APIMs model associations between one's own predictor and outcome (i.e., actor effects) and associations between one's own predictor and one's partner's outcome (i.e., partner effects). The APIMs in the current study were distinguishable by partner role, namely veteran or significant other. Our models included veterans' self‐reported PCL‐5 scores and significant others' collateral‐reported PCL‐5‐C scores as predictors and each partner's relationship satisfaction score (CSI‐32) as outcomes. Models also included the two variables capturing the magnitude (absolute difference) and direction (coded as 1 if veteran's score was higher, 0 if significant other's score was higher) of discrepancy between self‐ and collateral‐rated PTSD symptoms, as well as covariates of relationship length and respondent gender. All predictors were grand‐mean centered. Thus, the APIM framework provided a way to model the effects of discrepancy magnitude and direction while controlling for the overall level of PTSD symptoms.

**FIGURE 1:**
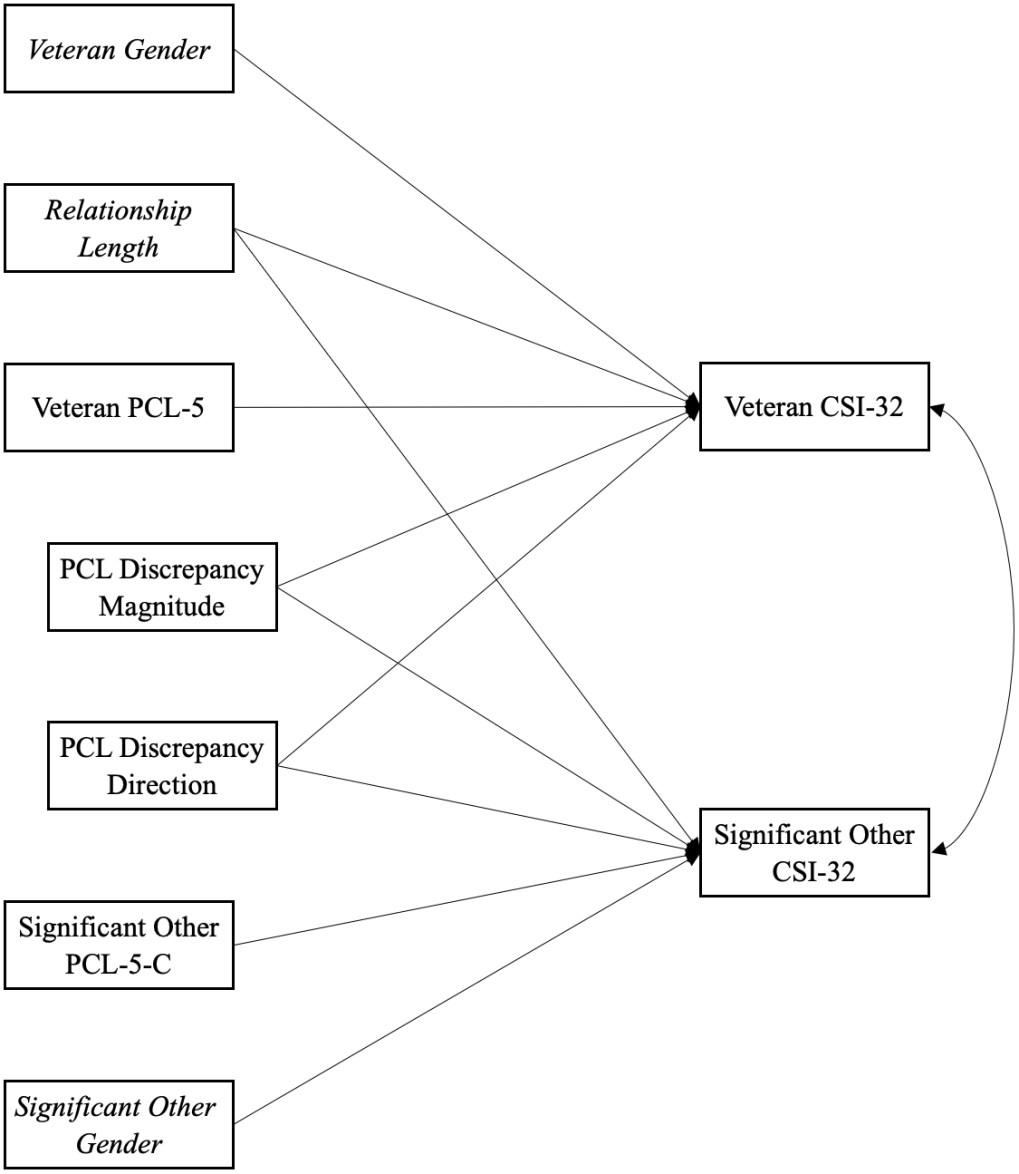
Standardized Path Estimates for APIM predicting Relationship Satisfaction (Model 1). All covariances between predictors were included, but they are not depicted here for simplicity. Italicized text denotes covariates. CSI‐32, Couples Satisfaction Index‐32; PCL‐5, PTSD Checklist for DSM‐5; PCL‐5‐C, Collateral‐reported PTSD Checklist for DSM‐5.

Following guidance from Fitzpatrick et al. (2016), preliminary analyses of the basic APIM (veteran‐ and significant other‐reported PTSD symptoms, relationship satisfaction, and covariates; no discrepancy variables) indicated an actor‐only pattern: partner effects were nonsignificant, and the *k* parameters, which capture the relative strength of actor and partner effects, showed a bootstrapped 95% confidence interval (CI) that overlapped with 0; *k*_veterans_ = 0.29 [−7.64, 4.52], *k*_partners_ = −0.18 [−1.14, 1.44]. This is consistent with other literature that has found partner effects to be substantially smaller than actor effects across many domains of relationship functioning (Joel et al., 2020). Thus, partner effects were dropped from the final APIMs. We ran five actor‐only APIMs: one using the total PTSD score and one for each of the four symptom cluster subscales. See Figure 1 for an example model. APIMs were run as structural equation models in the R software package lavaan (R Core Team, 2020; Rosseel, 2012) following analysis code from Stas et al. (2018), and were estimated using full information maximum likelihood estimation to account for missing data. All 199 couples were included in this analysis.

## RESULTS

Correlations between study variables are shown in Table 2. Bivariate correlations showed that veterans' and significant others' ratings of PTSD symptoms and relationship satisfaction were significantly correlated, as expected. Significant others showed a significant negative correlation between their ratings of veterans' PTSD symptom severity and their own relationship satisfaction. The magnitude of the discrepancy between each partner's PTSD symptom ratings was significantly and negatively correlated with PTSD symptom severity as rated by significant others but not veterans; in other words, when significant others perceived less severe PTSD, the discrepancy magnitude increased. Discrepancy magnitude was also significantly correlated with the direction of the discrepancy. The magnitude of the discrepancy was greater when the veteran's PTSD symptom ratings were higher than the significant other's collateral PTSD score.

**TABLE 2:** Correlations between study variables.

	1	2	3	4	5	6	7	8
1. Veteran PCL‐5								
2. SO PCL‐5‐C	0.278***							
3. PCL‐5 Discrepancy Magnitude	−0.013	−0.513***						
4. PCL‐5 Discrepancy Direction	0.441***	−0.534***	0.309***					
5. Veteran CSI	−0.129	−0.059	−0.048	0.026				
6. Significant other CSI	−0.038	−0.278***	0.030	0.134	0.526***			
7. Relationship Length	−0.119	−0.001	0.064	0.034	0.139	0.034		
8. Veteran Gender	0.081	0.016	−0.010	−0.039	0.070	0.153*	−0.177*	
9. SO Gender	−0.019	0.030	−0.069	0.005	0.015	−0.130	0.145*	−0.782***

*Notes*: PCL‐5 Discrepancy Magnitude is the log‐transformed absolute difference between Veteran PCL‐5 and SO PCL‐5‐C. PCL‐5 Discrepancy Direction is coded as 0 = SO's PCL‐5‐C score is higher and 1 = Veteran's PCL‐5 score is higher. Gender is coded as 0 = Male and 1 = Female.

Abbreviations: PCL‐5, PTSD Checklist for DSM‐5; PCL‐5‐C, Collateral‐reported PCL‐5; SO, significant other.

* *p* < 0.05;

*** *p* < 0.001.

### Aim 1: Evaluating agreement between veterans and significant others on PTSD symptom severity

One hundred seventy dyads completed both the PCL‐5 and PCL‐5‐C. Table 3 shows that the mean difference between the PCL‐5 (*M* = 49.46, *SD* = 15.24) and PCL‐5‐C (*M* = 39.22, *SD* = 20.18) was 10.25 points (*SD* = 21.64; *t*(169) = 6.18, *p ≤ 0*.001). The range of discrepancies between the two measures was quite large (−42 to 62), suggesting considerable variability in this sample. For a majority of couples (65.3%), veterans' PTSD symptom severity scores were greater than or equal to their significant other's ratings. Quartile differences were − 5.25 (25th percentile), 10.00 (50th percentile), and 27.00 (75th percentile). Large discrepancies also existed for each of the four symptom cluster subscales, with veterans endorsing more symptoms than their significant others on average for each symptom cluster; see Table 3. Intraclass correlations were low for PCL‐5 and PCL‐5‐C. Table 3 shows that the ICC for total score on the PCL and PCL‐5‐C was 0.375 with a 95% confidence interval from 0.136 to 0.546. Subscale ICCs ranged from 0.150 to 0.455.

**TABLE 3:** PTSD checklist (PCL‐5 or PCL‐5‐C) total and subscale scores, intraclass correlation coefficients, range, and mean difference scores.

Scale or subscale	Range	Veteran		Significant other		ICC	Veteran‐SO range		Veteran‐SO difference	
		M	SD	M	SD		Min	Max	M	SD
Total	0–80	49.46	15.24	39.21	20.18	0.375	−42	62	10.25	21.64
Intrusions	0–20	12.09	4.55	9.96	5.75	0.280	−14	18	2.13	6.66
Avoidance	0–8	5.58	2.13	4.28	2.33	0.157	−5	8	1.31	2.99
Negative alterations in cognition and mood	0–28	16.56	6.25	13.31	8.13	0.450	−17	22	3.25	8.65
Alterations in arousal and reactivity	0–24	15.11	5.10	11.64	6.36	0.427	−16	19	3.47	6.69

Abbreviation: ICC, intraclass correlation coefficient; SO, significant other.

### Aim 2: Evaluating associations between PTSD Total scores and subscale scores, symptom agreement, and relationship satisfaction

Table 4 shows standardized path coefficients for all estimated paths in each of the five actor‐only APIMs. For total PCL‐5 score, veterans' self‐reported PTSD symptoms were significantly and negatively associated with their relationship satisfaction. Discrepancy magnitude and direction variables were both nonsignificant. For significant others, total collateral PTSD score and discrepancy magnitude were both significantly and negatively associated with relationship satisfaction. Direction of discrepancy was nonsignificant.

**TABLE 4:** Standardized path coefficients from actor‐partner interdependence models for PCL‐5 total score and four subscales predicting relationship satisfaction.

	PCL total		Intrusion		Avoidance		Negative cog/mood		Hyperarousal	
	β [95% CI]	*p*	β [95% CI]	*p*	β [95% CI]	*p*	β [95% CI]	*p*	β [95% CI]	*p*
Veteran effects										
PTSD (PCL‐5)	−0.223 [−0.369, −0.076]	0.003	−0.079 [−0.229, 0.070]	0.299	−0.089 [−0.263, 0.085]	0.315	−0.268 [−0.402, −0.133]	<0.001	−0.111 [−0.255, 0.033]	0.132
Discrepancy Magnitude	−0.103 [−0.258, 0.052]	0.194	−0.011 [−0.158, 0.135]	0.878	0.029 [−0.119, 0.178]	0.700	−0.137 [−0.282, 0.008]	0.064	−0.116 [−0.267, 0.034]	0.130
Discrepancy Direction	0.165 [−0.002, 0.332]	0.052	0.068 [−0.091, 0.228]	0.402	−0.008 [−0.196, 0.179]	0.930	0.161 [0.007, 0.314]	0.040	0.086 [−0.073, 0.245]	0.288
Relationship Length	0.157 [0.011, 0.304]	0.036	0.154 [0.003, 0.304]	0.045	0.159 [0.010, 0.309]	0.037	0.162 [0.018, 0.306]	0.028	0.160 [0.013, 0.308]	0.033
Gender	0.096 [−0.038, 0.230]	0.160	0.090 [−0.047, 0.228]	0.196	0.088 [−0.048, 0.224]	0.204	0.104 [−0.029, 0.237]	0.125	0.072 [−0.063, 0.208]	0.296
Significant other effects										
PTSD (PCL‐5‐C)	−0.345 [−0.511, −0.179]	<0.001	−0.209 [−0.383, −0.036]	0.018	−0.207 [−0.388, −0.026]	0.025	−0.353 [−0.514, −0.193]	<0.001	−0.209 [−0.377, −0.040]	0.015
Discrepancy Magnitude	−0.185 [−0.350, −0.019]	0.029	−0.026 [−0.180, 0.127]	0.735	−0.075 [−0.243, 0.093]	0.382	−0.148 [−0.294, −0.002]	0.047	−0.217 [−0.369, −0.066]	0.005
Discrepancy Direction	0.048 [−0.114, 0.210]	0.565	0.049 [−0.126, 0.224]	0.585	−0.008 [−0.181, 0.164]	0.924	0.058 [−0.107, 0.223]	0.492	0.123 [−0.040, 0.286]	0.140
Relationship Length	0.054 [−0.083, 0.191]	0.437	0.045 [−0.095, 0.185]	0.526	0.057 [−0.085, 0.199]	0.430	0.038 [−0.097, 0.173]	0.581	0.042 [−0.096, 0.180]	0.549
Gender	−0.168 [−0.292, −0.045]	0.008	−0.180 [−0.306, −0.054]	0.005	−0.167 [−0.294, −0.039]	0.010	−0.142 [−0.265, −0.020]	0.023	−0.147 [−0.273, −0.022]	0.022

*Notes*: For discrepancy direction, higher scores for veterans is represented by a positive value, higher score for the significant other are represented by a negative value. For gender, negative values represent higher scores for males.

Abbreviations: CI, confidence interval; PCL‐5, PTSD Checklist for DSM‐5; PCL‐5‐C, Collateral‐report PTSD Checklist for DSM‐5.

For the symptom clusters of intrusion and avoidance, no associations with PTSD predictors were significant for veterans. Collateral PCL ratings of intrusion and avoidance severity were significantly and negatively associated with relationship satisfaction for significant others, but discrepancy direction and magnitude were not.

For the symptoms cluster of negative cognition and mood, both veterans' self‐reported symptom severity and the direction of the discrepancy were significantly associated with relationship satisfaction. Veterans reported higher relationship satisfaction when their negative cognition and mood symptoms were lower but also when they reported more severe cognition and mood symptoms than their significant others reported. For significant others, both the collateral‐report cognition/mood symptom severity and the discrepancy magnitude were significantly and negatively associated with relationship satisfaction. More severe perceived mood/cognition symptoms and a larger discrepancy between the two partners' scores both predicted lower relationship satisfaction for significant others.

For the symptom cluster of hyperarousal, there were no significant effects for veterans. Significant others' collateral‐reported hyperarousal symptom severity and the discrepancy magnitude showed negative associations with significant others' relationship satisfaction, such that more severe hyperarousal symptoms and a larger discrepancy between partners' scores were associated with lower relationship satisfaction.

Relationship length and gender were both included in the models as covariates. For veterans but not significant others, relationship length was associated with satisfaction, with longer relationships associated with greater satisfaction in all models. For significant others but not for veterans, gender predicted satisfaction in all models, with higher satisfaction for significant others who identified as male.

## DISCUSSION

Results from the current study are consistent with the existing body of research indicating that higher veteran self‐reported and higher significant other‐perceived PTSD severity is associated with worse relationship satisfaction for both partners, particularly in terms of negative cognition/mood and hyperarousal symptoms. A number of prior studies have shown that the negative cognitions and mood cluster (referred to as the emotional numbing cluster prior to DSM‐5) is the PTSD symptom cluster most strongly related to relationship functioning (Renshaw & Caska, 2012), and arousal and reactivity symptoms tend to be associated with relationship conflict and aggression (Allen et al., 2018). Our findings are also consistent with previous research which suggests that relationship partners differ in their understanding of the presence and severity of PTSD symptoms. This study contributes new information about how discrepancies in veterans' and significant others' perceptions of veterans' PTSD symptoms are related to relationship functioning, and how the implication of these discrepancies may differ for veterans and their intimate partners. Specifically, these findings illustrate a key feature of the cognitive‐behavioral interpersonal model of PTSD: how each partner's respective interpretation of symptoms is related to satisfaction, an aspect of the overall relationship milieu (Monson et al., 2010).

Overall, results from this study showed that veterans and their significant others had substantial discrepancies in their observation and interpretation of veterans' PTSD symptoms. Correlations showed that couples evidenced larger magnitude discrepancies in their PTSD and when the discrepancy was in the direction of significant other‐rated scores being lower than veteran‐rated scores. Taken together, this indicates that greater discrepancies occur when significant others perceive fewer PTSD symptoms. This may be interpreted as a tendency for intimate partners to under‐perceive symptoms or to fail to attribute observed behaviors or emotional states correctly to PTSD symptoms. Significant others interpret PTSD symptoms through observation and communication, which can be influenced by many different factors, including overall understanding of PTSD. Internalizing symptoms of PTSD (e.g., avoidance, numbing, and anger) can create confusion as they often present in non‐specific ways and can lead to communication difficulties, missed opportunities to connect, and withdrawal. This may leave the significant other with an understanding that emotional distress is present for the veteran, but with a limited understanding of how this connects specifically to PTSD (Barry et al., 2019; Sommer et al., 2019).

At the same time, veteran self‐reported symptoms are not necessarily more “accurate” than significant others'. Veterans may have difficulty understanding, and expressing their own symptoms. There are also many factors that could influence how veterans complete self‐report questionnaires. Indeed, some research suggests patient‐reported PCL scores may capture experiences beyond PTSD symptoms, including trait neuroticism, misunderstanding of symptom terminology, and general distress beyond the timeframe of assessment that is not specifically anchored to a traumatic experience (Kramer et al., 2023).

This study is the first we know of to show that the discrepancy between PTSD‐diagnosed veterans and their significant others' reports of PTSD symptoms, independent of the severity of symptoms, is associated with poorer relationship functioning. For significant others, larger magnitude of discrepancies between their assessment of PTSD severity and their veteran's report of PTSD severity predicted lower relationship satisfaction, even after controlling for the overall PTSD severity level. Symptom cluster analyses showed that this finding was largely driven by the discrepancies in the negative cognition and mood cluster (e.g., anhedonia and isolation) and the hyperarousal cluster (e.g., anger/irritability and sleep problems). In other words, significant others experienced poorer relationship satisfaction when they did not perceive the same level of negative cognition and mood and hyperarousal symptoms that the veterans themselves reported, above and beyond the lower satisfaction attributable to more severe PTSD symptoms in general. Presumably, intimate partners may be noting some negative behaviors or emotional states in their PTSD‐diagnosed partners that are associated with poorer relationship satisfaction, but are not attributing these behaviors and emotions to PTSD symptoms.

These findings contribute to a larger understanding of PTSD in a relational context and expand the work by Renshaw and Caska (2012), who found that partners' attributions regarding the cause and nature of veterans' PTSD symptoms are associated with the intensity of the distress ratings they assign to symptoms. In general, attibutions in relationships are influenced by the quality of the relationship (e.g., behaviors are interpreted more negatively in distressed couples and more positively or neutral in more satisfied couples); over time, these interpretations could either strengthen (positive attributions) or weaken (negative attributions) the relationship (Bradbury & Fincham, 1992). These results are based on cross sectional data, so it is impossible to determine a causal relationship or the influence of these phenomena over time. On one hand, it is possible that the connection between symptom report discrepancies and relationship satisfaction is an indication that significant others make attributions about PTSD symptoms that could contribute to relationship erosion. Negative cognition and mood are internalized (intrapersonal) rather than externalized (behavioral or interpersonal) symptoms. It may be particularly difficult for significant others to have an understanding of veterans' cognitive and mood symptoms as they occur in the veteran's internal world. Without adequate communication from the veteran to the significant other to share or report those experiences, there would be no way for the significant other to access the extent of those symptoms. As a result, significant others could make negative interpretations of symptoms like irritability or avoidance when they impact the relationship. Over time, the result could be frustration and poorer relationship quality. Significant others may also report less negative ratings of relationship functioning when they attribute their partners' hyperarousal symptoms (e.g., vigilance and irritability) to PTSD rather than personalizing or globalizing them.

On the other hand, these findings could suggest that less satisfied significant others are also less attuned to their veteran partner's distress, leading to a discrepant symptom assessment. Lower satisfaction and higher distress may make it more difficult for individuals to recognize and communicate about symptoms, leading to higher discrepancies between veterans and their significant others. Previous findings have highlighted the important bidirectional and reciprocal links between dysfunctional communication and PTSD symptom ratings (Fredman et al., 2017), so a unidirectional interpretation of the current findings is almost certainly incomplete.

For veterans, the direction of the discrepancy between significant other and veteran reports of PTSD symptom severity, rather than the magnitude, predicted lower relationship satisfaction above and beyond the severity of PTSD symptoms. This finding was largely driven by the negative cognition and mood cluster (e.g., anhedonia and isolation): veterans reported higher relationship satisfaction when they reported more severe negative cognition and mood symptoms than their significant others perceived, regardless of how severe the cognition and mood symptoms were. This is a somewhat puzzling finding in contrast with the results for significant others, which suggests that significant others' individual and relational wellbeing may benefit from perceiving more of the veteran's PTSD symptoms. Here, it seems that significant others perceiving fewer symptoms of negative cognitions and mood may be protective for veterans. It may be beneficial for relationships if veterans interact with their significant others with a more positive mindset and mood than they actually feel. Alternatively, it could be that veterans in happier relationships feel better when interacting with their significant others, so negative cognition and mood symptoms are not as apparent to significant others. This finding may also reflect an attempt at protective buffering, which refers to one partner attempting to “protect” the other by withholding potentially stressful information (Joseph & Afifi, 2010). In the case of PTSD, this could mean a veteran withholding information on traumatic experiences during their deployment or choosing to not discuss current PTSD symptoms and associated distress. However, protective buffering is typically associated with increased distress and lower relationship satisfaction in the partner who is engaging in the protective buffering efforts (Carter et al., 2020). More research is needed to replicate and explain this finding and understand whether and under what conditions PTSD‐diagnosed patients may benefit from their significant others being more aware of their symptoms.

It is also notable that, regarding overall links between PTSD symptom severity and relationship satisfaction, our results showed the strongest and most consistent associations for significant others rather than for veterans. Significant others' relationship satisfaction was related to their collateral PTSD reports for the total PTSD score and all four symptom clusters, while veterans' relationship satisfaction was only related to the negative cognition and mood cluster. This fits with a growing body of evidence arguing for the importance of including significant others in veterans' PTSD care. Fostering a shared understanding of PTSD and communication strategies for managing day‐to‐day interactions in the context of PTSD could improve relationship satisfaction over time.

The benefits of a shared understanding of PTSD treatment are supported by some literature on couples‐based interventions for PTSD. For example, Structured Approach Therapy (Sautter et al., 2016) cites empathic communication as a mechanism of action whereby couples build a shared awareness of symptoms using disclosure of experiences and discussion of symptoms. Balderrama‐Durbin et al. (2013) also found that combat disclosure mediates the effects of PTSD on relationship satisfaction, suggesting that higher partner support results in a safer context for trauma disclosure, which could reduce the impact of combat exposure.

Finally, relationship length and gender were included as control variables rather than focal variables of interest. While we did not have specific hypotheses about their effects, longer relationship length was consistently related to higher relationship satisfaction for veterans, even controlling for the severity of PTSD and PTSD‐reporting discrepancies. One of the hallmark features of PTSD is a sense of foreshortened future and pessimism about future events. A committed, long‐lasting relationship may combat negative trauma‐related beliefs about the future, which may be one mechanism by which a long‐standing relationship can buffer against PTSD. However, PTSD symptoms also exert a deleterious impact on relationship functioning, undermining this positive influence over time. Thus, helping couples strengthen their relationship is an important intervention target for partnered veterans. Additionally, a majority of significant others (84%) were female whereas the majority of veterans (83%) were male. Renshaw et al. (2014) found that female veterans and female significant others of veterans showed a stronger link between the emotional numbing symptom cluster and relationship dysfunction than male veterans or significant others. Research also suggests that women may be more negatively impacted by power imbalances in relationships (Sprecher & Felmlee, 1997). Thus, women, whether veterans or significant others of veterans, may be more attuned to and affected by discrepancies in perspectives within a relationship.

On the whole, these findings speak to the value of couple‐based PTSD interventions that seek to improve both individuals' understanding of PTSD symptoms. CBCT (Monson et al., 2011; Monson et al., 2012) and SAT (Sautter et al., 2015) aim to increase communication about PTSD‐related thoughts and emotions, jointly approach and challenge avoided thoughts and behaviors, and actively solve problems together. PTSD Family Education (PFE; Sautter et al., 2015) focuses on increasing shared understanding by providing information about PTSD to patients, significant others, and family members together. We found substantial discrepancies in PTSD symptom reports between partners in a couple, which seems potentially related to intimate partners not attributing observed emotions or behaviors to PTSD and/or to veterans not fully recognizing, interpreting, or sharing their internal experiences. At least for intimate partners, this discrepancy was connected to poorer relationship satisfaction. Fredman et al. (2017) discuss the importance of identifying “modifiable factors” that can be targeted in treatment to improve symptoms. Future research could focus on whether bringing views of PTSD into alignment through psychoeducation and experiential exercises could represent a specific, modifiable way that treatment could potentially reduce overall distress and improve relationship functioning. It may be the case that discrepant views of PTSD cause relationship problems, or it may be the case that distress causes poor understanding between veterans and significant others, which then leads to discrepancies in reported scores, or it could be bidirectional. Regardless of the direction of influence, treatments that target relationship functioning and PTSD symptoms concurrently are likely useful.

There are limitations to the current study that are important to consider. As previously discussed, our data are correlational. Future research will need to utilize longitudinal and interventional methods to disentangle directional and causal relationships between PTSD symptom discrepancies and distress. Second, our sample was selected from dyads seeking a couples‐based treatment for PTSD at a VA medical center. Participants may not be representative of all veteran couples with PTSD, let alone non‐veteran couples with PTSD. Dual PTSD couples and couples with the poorest relationship functioning and highest risk (e.g., suicidality, severe substance use, and moderate to severe domestic violence) who were excluded prior to the baseline assessment (e.g., during phone screen, etc.) given contraindications for the RCT. Veterans seeking PTSD treatment in VA settings may also experience more clinical comorbidities, overall distress, and treatment‐resistant symptoms than other veterans. Third, the PCL‐5‐C has not been widely studied and, as such, the psychometric properties of the PCL‐5‐C are not well‐established. In this sample, internal consistency was good, but there may be other issues that could impact the validity of these discrepancy scores. Additionally, the avoidance subscale included only two items for each partner. This may have restricted the range of discrepancy scores and potentially underestimated the effect of these differences in reported symptom severity. Fourth, the original clinical trial was not specifically powered for this secondary analysis, and thus insufficient statistical power could explain any null findings. Finally, this analysis focused specifically on veterans' PTSD symptoms and did not address significant others' distress. Future research could evaluate the impact of significant others' own distress on their perceptions of PTSD in their veteran partner, as well as the impact of discrepancy on significant others' individual well‐being.

In spite of these limitations, this study shows that discrepancies in conjoint perceptions of PTSD symptoms may have important implications that warrant further study. In particular, couple‐based PTSD intervention studies will provide clinically relevant data on the causal link between PTSD report discrepancies and distress, as well as whether discrepancies might change over time. For example, as the couple completes treatment, do their perceptions of symptoms converge? Does this increased alignment predict improvements in relationship satisfaction and functioning over time? In addition, more work is needed to clarify the mechanisms by which couples experience discrepancy versus alignment in the perception of symptoms, as well as the mechanisms of connection between discrepancies and distress. Communication, intimacy, and cognitive attributions may all play important roles, but additional research is needed to disentangle these explanations.

## FUNDING INFORMATION

This study is a secondary analysis of data collected in a CBCT trial, funded by Award Number 1I01RX002093‐01 from the VA Office of Research and Development, Principal Investigator is Leslie Morland. The datasets analyzed during the present study are not publicly available. Study materials are available from the principal investigator upon reasonable request.
